# Pacing, Conventional Physical Activity and Active Video Games to Increase Physical Activity for Adults with Myalgic Encephalomyelitis/Chronic Fatigue Syndrome: Protocol for a Pilot Randomized Controlled Trial

**DOI:** 10.2196/resprot.7242

**Published:** 2017-08-01

**Authors:** Katia Elizabeth Ferrar, Ashleigh E Smith, Kade Davison

**Affiliations:** ^1^ Alliance for Research in Exercise, Nutrition and Activity School of Health Sciences University of South Australia Adelaide Australia

**Keywords:** fatigue syndrome, chronic, video games, exercise

## Abstract

**Background:**

Myalgic encephalomyelitis/chronic fatigue syndrome (ME/CFS) is a serious illness of biological origin characterized by profound physical and cognitive exhaustion and postexertion malaise. Pacing is a common strategy used to manage available energy and complete activities of daily living; yet little research has investigated this as a strategy to increase physical activity levels. Typically, people living with ME/CFS are faced by unique barriers to physical activity participation and are less physically active than healthy peers. As such they are at increased risk of physical inactivity–related health consequences. Active video games may be a feasible and acceptable avenue to deliver physical activity intervention by overcoming many of the reported barriers to participation.

**Objective:**

The primary objective of this pilot study is to determine the feasibility and acceptability of active video games to increase physical activity levels of people with ME/CFS. The secondary aims are to explore the preliminary effectiveness of pacing and active video gaming to pacing alone and pacing plus conventional physical activity to increase the physical activity levels of adults with ME/CFS and explore the relationship between physical activity and cumulative inflammatory load (allostatic load).

**Methods:**

This study will use a mixed method design, with a 3-arm pilot randomized controlled trial, exit interviews, and collection of feasibility and process data. A total of 30 adults with ME/CFS will be randomized to receive either (1) pacing, (2) pacing and conventional physical activity, or (3) pacing and active video gaming. The intervention duration will be 6 months, and participants will be followed up for 6 months postintervention completion. The intervention will be conducted in the participant’s home, and activity intensity will be determined by continuously monitored heart rate and ratings of perceived exertion. Feasibility and acceptability and process data will be collected during and at the end of the intervention. Health-related outcomes (eg, physical activity, blood samples, quality of life, and functioning) will be collected at baseline, end of intervention, and 6 months after intervention completion.

**Results:**

This protocol was developed after 6 months of extensive stakeholder and community consultation. Enrollment began in January 2017; as of publication, 12 participants were enrolled. Baseline testing is scheduled to commence in mid-2017.

**Conclusions:**

This pilot study will provide essential feasibility and acceptability data which will guide the use of active video games for people with ME/CFS to increase their physical activity levels. Physical activity promotion in this clinical population has been poorly and under-researched, and any exploration of alternative physical activity options for this population is much needed.

**Trial Registration:**

Australia New Zealand Clinical Trials Registry: ACTRN12616000285459; https://www.anzctr.org.au/Trial/Registration/TrialReview.aspx?id=370224 (Archived by WebCite at http://www.webcitation.org/6qgOLhWWf)

## Introduction

Myalgic encephalomyelitis/chronic fatigue syndrome (ME/CFS) is a complex, serious illness of biomedical origin characterized by profound physical and cognitive exhaustion and postexertion malaise [1]. Symptoms such as fatigue and cognitive impairments are typically made worse to the point of collapse after physical exertion [1]. The combination of physical inability to be active due to the condition and behaviors to avoid potential flare-up results in people with ME/CFS being less physically active than healthy controls [2].

The mechanisms underlying ME/CFS remain unclear, and studies report associations with multiple individual proinflammatory markers and indicators of immune activity, but the findings are inconsistent [3]. A potential mechanism by which physical activity plays a role in managing chronic conditions is through allostasis: the body’s response to stress orchestrated by the hypothalamic-pituitary-adrenal axis and the central nervous system via the production of stress hormones [4]. The detrimental negative effect of allostasis can be quantified as allostatic load (AL). The theoretical mechanism between AL and physical activity has recently been empirically supported, with higher levels of physical activity associated with lower AL in Mexican American adults [5] and middle-aged women [6]. Further, adults living with ME/CFS are reported to have higher AL than healthy controls [7,8]. It is widely accepted that cumulative biomarkers are stronger predictors of morbidity and mortality, yet recent research into ME/CFS continues to investigate individual neuroendocrine and immune biomarkers with limited success and inconsistent findings [3]. Further, few AL studies are longitudinal or interventional in nature, limiting the relationships and conclusions that can be drawn.

Physical activity is essential for health and well-being and has a role in prevention and management of muscle atrophy and many chronic conditions such as cardiovascular disease, osteoporosis, and some cancers [9]. There is much debate in the literature surrounding the effectiveness and safety of exercise or physical activity-based interventions for people with ME/CFS. In this population, the majority of physical activity–based interventions have employed a Graded Exercise Therapy (GET) approach. Typically, after setting baseline levels, the intention of GET protocols is to incrementally increase activity despite possible changes in symptom severity. One common criticism of GET protocols is they typically suggest stabilization of activity progression at the point of symptom flare-up but do not appear to include modification or cessation of activity to promote symptom resolution. The results of GET protocols are mixed, with some reporting symptom improvement [10,11]. The risk of adverse responses to GET is not clear from intervention studies; however, support group surveys of people living with ME/CFS suggest a significant proportion of these interventions result in negative experiences during the intervention [12].

An alternative approach to delivery of physical activity interventions is pacing. Pacing is a typical strategy used by people with ME/CFS to manage their activities of daily living within their available energy envelope by structuring and organizing their daily activities. Pacing is typically well accepted by people living with ME/CFS (according to patient surveys) and is used primarily to manage symptom flare-ups and work within the available energy, but it is also recommended that pacing can help to slowly increase the energy envelope and thus the level of activity performed. Pacing is a flexible, symptom-contingent process that can be used to increase physical activity levels. Survey data suggests a greater percent of people living with ME/CFS feel pacing improved their symptoms (45%, n=69) compared to GET (12%, n=77) [12]. In addition, anecdotal evidence suggests a large percentage of people living with ME/CFS already use pacing and heart rate (HR) monitoring to some extent (personal communication by ME/CFS Australia [SA] spokesperson, March 2016), and as such, pacing will be considered the control situation for this study.

Only one published study can be located that has employed a flexible symptom-contingent protocol (ie, pacing using HR and perceived exertion monitoring) to increase activity levels in people with ME/CFS [13]. The study demonstrated improvements in physiological outcomes (eg, blood pressure, cognitive ability) but no change in physical activity between the groups postintervention. The study used a self-report activity questionnaire with poor reliability at low intensity levels of activity, which is concerning because this accounts for almost all physical activity in most people with or without ME/CFS, and only low to moderate validity with other self-reported physical activity outcomes [14]. Overall, the flexible, symptom-contingent approach to increasing activity levels in people with ME/CFS has not been adequately researched. An important finding from the Wallman et al [13] study is that none of the participants in the exercise group rated themselves as any worse and 91% rated themselves some degree better overall. The current study will build on this small body of literature by investigating pacing alone versus pacing and physical activity using objective measures of physical activity and safely monitoring activity intensity via continuous HR monitoring and ratings of perceived exertion (RPE).

People with ME/CFS report unique barriers to participation in physical activity. These barriers include extreme fatigue just getting to and from structured activity, greater difficulty being active standing compared to sitting, and ability to participate in only very short durations of activity [1]. Interventions using commercially available active video games, such as Xbox Kinect, can overcome many of these barriers for people with ME/CFS. Active video games can be played at home either while standing or seated, can be easily played for short periods of time (eg, 1-2 minutes), and provide choice of different gaming activities—factors which will potentially reduce patient dissatisfaction and drop out [15].

An inability to fully participate in their family’s lives and community often results in people living with ME/CFS reporting depression, social isolation, and lack of connectedness [1]. Active video games have the potential to provide an avenue for family connectedness and promote a feeling of normality while enabling participation (potentially with others) in an everyday activity. People’s experiences with active video gaming suggest substantial benefits including feelings of empowerment, reductions in depression, increased psychosocial well-being, and increased social connectedness by playing with family members [16].

Active video gaming has been shown to be feasible and acceptable to other chronic health populations such as those with cerebral palsy and stroke and is commonly played while sitting in these populations [17]. Evidence suggests active video gaming can improve a range of rehabilitative outcomes, increase physical activity levels among sedentary and frail elderly adults, and increase cognitive performance and reduce fatigue in women with systemic lupus erythematosus [18,19,20]. Despite the evidence to suggest active video gaming as effective and relevant, no studies have investigated the feasibility or acceptability of active video gaming in adults with ME/CFS.

The primary objective of this pilot study is to investigate the feasibility and acceptability of pacing and active video gaming to increase the physical activity levels of adults with ME/CFS.

The secondary objectives of this study are to (1) explore the preliminary effectiveness of pacing and active video gaming to pacing alone and pacing and conventional physical activity to increase the physical activity levels of adults with ME/CFS and (2) explore the relationship between allostatic load and physical activity in people with ME/CFS.

The related hypotheses are (1) preliminary evidence of benefit from pacing and active video gaming to increase physical activity compared to pacing alone will be demonstrated, (2) preliminary evidence of equal benefit of pacing and video gaming to increase physical activity levels compared to pacing and conventional exercise will be demonstrated, and (3) preliminary evidence of a negative relationship will be demonstrated between allostatic load and physical activity.

## Methods

### Trial Design

A mixed-method study will be conducted comprising an exploratory 3-armed randomized controlled pilot study (1:1:1 allocation ratio) of pacing versus pacing and conventional physical activity versus pacing and active video gaming; qualitative exit interviews and collection of process data to investigate the feasibility and acceptability of the approach. The study protocol has been approved by the University of South Australia Human Research Ethics Committee (protocol 0000035299). The protocol is registered with the Australia New Zealand Clinical Trials Registry [ACTRN12616000285459]. All participants will be required to provide written informed consent prior to enrolling in the study and report general practitioner consent to participate.

### Protocol Development

A stakeholder advisory group was convened including 2 representatives from the local state ME/CFS society, a rheumatologist with extensive clinical and research knowledge of ME/CFS, a general physician with extensive clinical expertise in managing ME/CFS, 2 adults currently living with ME/CFS, and the research team. The primary purpose of the stakeholder advisory group was to assist in the consumer-driven and evidence-based development of the final protocol. A 6-month consultative development phase was completed in December 2016. The stakeholder advisory group welcomed and considered comments and concerns from external bodies (eg, national and international support groups).

### Intervention

**Figure 1:**
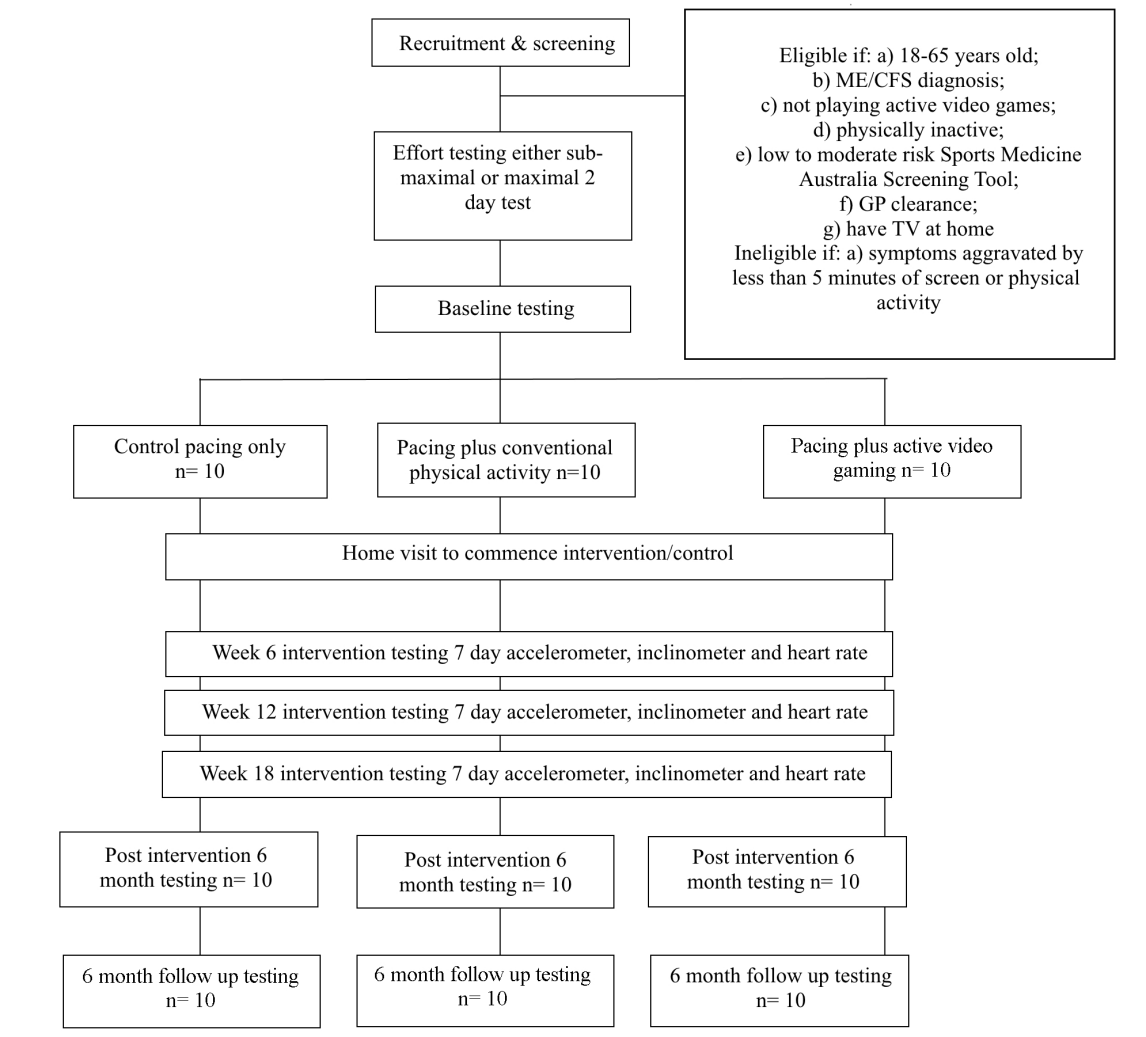
Study Flow Chart.

After baseline assessment, participants will be block randomized (block size = 6, 1:1:1 allocation ratio) into pacing only, pacing plus conventional physical activity, or pacing plus active video gaming (Figure 1).

Participants will be randomized using a computer-generated random number sequence and opaque sealed envelopes generated by a University of South Australia researcher not involved in the study. All participants will receive telephone support from the research assistant once a week for the first 3 months of the intervention and every 2 weeks for the last 3 months of the intervention, and participants can contact the research assistant at any additional time. At the end of the 12 months, participants still enrolled will be provided with an Xbox Kinect unit in acknowledgement of their participation.

### Control Condition

In the pacing only condition, participants will be educated about pacing to manage their daily energy levels and informed not to commence a physical activity program for 6 months. Pacing is a process whereby activities of daily living are organized and timed in order to not exceed the available energy for that particular day. Pacing will be self-monitored to avoid excessive exertion by means of continuous HR monitoring and RPEs maintained below a designated threshold. The HR and RPE thresholds will be determined during the baseline effort test, which is described in detail in the Methods section. Participants in the pacing only control condition will be provided with no instruction to increase physical activity levels.

### Intervention Conditions

The intervention will be 6 months in duration. In the pacing plus conventional activity condition, participants will be educated about pacing and prescribed a home-based participant-controlled symptom-contingent physical activity program involving a conventional activity type selected by the participant such as walking, elastic band resistance exercise, or cycling.

In the pacing and active video gaming condition, participants will be provided with an Xbox Kinect (set up in their home) and prescribed a home-based participant-controlled symptom-contingent physical activity program involving dance and sport games (Sports Rivals and Dance Central). Swapping passive screen time (such as television viewing) with the active gaming will be recommended. If the participants report no or minimal screen time, they will be encouraged to swap out other activities that require low cognitive or physical load. The active video gaming and conventional physical activity is intended to be additional to existing physical activities.

Participants in the two conditions involving physical activity (ie, pacing and conventional physical activity and pacing and active video gaming) will have their starting physical activity prescription developed by the research assistant who will be an accredited exercise physiologist with at least 2 years of clinical experience. The research assistant will develop the prescription based on participant self-reported amount of activity and symptom relationship from the Symptom History Questionnaire (detailed later in Methods section). Prescription will involve allocation to 1 of 3 tiers: light tolerance, moderate tolerance, or high activity tolerance (Table 1).

**Table 1:** Physical activity prescription triage categories.

Activity tolerance tier	Categorization	Baseline	Progress
Exclude	Self-reported flare-up of symptoms with less than 5 minutes of screen time; moderate to severe symptom flare-up with less than 5 minutes of self-reported light physical activity or household chores	—	—
Light	Flare-ups with predominantly moderate to high intensity tasks or longer bouts of light intensity tasks or pushing energy envelope with too many activities on a given day; able to participate in light activity for at least 5 minutes (but less than 10 minutes)	2 minutes per day (6-8 minutes per week)	30 seconds to 1 minute per day per intervention week
Moderate	Symptom flare-ups with activities predominantly considered moderate or high intensity (ie, minimal flare-ups with light intensity tasks), able to be active for between 10 to 15 minutes at a light intensity with minimal or no flare-up of symptoms	5 minutes per day (15-20 minutes per week)	1 to 2 minutes per day per intervention week
High	Reported symptom flare-ups with activities predominantly considered high intensity or longer bouts moderate tasks (ie, minimal flare-ups with light intensity and short bouts of moderate intensity tasks), able to be active for 15 or more minutes at a light intensity with minimal or no flare-up of symptoms	10 minutes per day (30-40 minutes per week)	2 to 4 minutes per day per intervention week

Physical activity will be prescribed every second day (at the most) and perceived exertion will be monitored by the participant remaining under the set HR and RPE thresholds at all times. Participants will be in full control of their program and will be able to stop physical activity sessions entirely; plateau; or reduce frequency, duration, or intensity of sessions to manage symptom occurrences. Activity sessions can be completed in one session (eg, 1 10-minute block) or split up throughout the day.

Any activity progression will occur every 2 weeks. To be eligible to progress, participants must demonstrate symptoms are stable. Stable equates to HR and RPE zones adhered to for the 2-week period and no increase in symptoms as a result of activity. Progression can be escalated (particularly as higher volumes demonstrated) with consultation with research team and research assistant only if symptoms are stable. The active gaming or conventional activity will be encouraged on those days that the participants consider there to be adequate energy to do all activities of daily living usual for that person. Participants will also be encouraged to consider cognitive activities as potentially draining in addition to physical activities.

### Setting

Participants will be recruited from Adelaide, South Australia, Australia. Testing will occur during face-to-face sessions at the University of South Australia, City East Campus, Adelaide, Australia. Home visits will be conducted to educate the participants regarding their condition allocation and set up video games where applicable, and the remainder of the intervention will be home-based.

### Participants

Inclusion criteria:

- Aged from 18 to 65 years
- General practitioner clearance
- Self-reported diagnosis of ME/CFS by a general practitioner or medical specialist based on one of the commonly accepted criteria (the Oxford Criteria is not acceptable)
- Able to complete exercise test (either maximal or submaximal)
- Not currently playing active video games
- Score low to moderate on Sports Medicine Australia Exercise Screening Tool [21]
- Self-report less than 150 minutes of moderate intensity activity each week (not meeting Australian National Physical Activity Guidelines for adults [22])
- Have a television in their home (Internet not required) and be willing to develop video game literacy

Exclusion criteria:

- Self-reported aggravation of symptoms with 5 or fewer minutes of screen time
- Self-reported aggravation of symptoms with 5 or fewer minutes of light intensity physical activity or movement

### Sample Size

This is the first study to investigate the feasibility and acceptability of active video gaming to increase the physical activity levels of people living with ME/CFS. A sample of 30 participants was deemed appropriate to provide the process feedback regarding testing procedures, and feasibility and acceptability data will be collected from 10 participants regarding the active video gaming experience.

### Recruitment

Participants will be recruited via local community support organizations, fliers, Facebook advertising, and the University of South Australia Clinical Trials Facility website.

### Feasibility and Acceptability

To explore the feasibility of implementation, total testing time will be calculated at each testing point for each participant. Statistics regarding the total amount and type of resources required (eg, consumables, printing) will be collected. Recruitment feasibility will be investigated after recruitment phase and involve analysis of recruitment statistics (eg, duration, ineligibility vs eligibility ratio number of recruitment avenues). Comprehensive informal exit interviews will be conducted with the active video game intervention group participants at the end of the intervention to explore acceptability either face-to-face or over the telephone. Information will be obtained on general satisfaction with the program, perceived cost effectiveness, acceptability of the testing protocols, and desire to continue with active video gaming.

### Effort Testing (Baseline Only)

Prior to randomization, participants will be screened for eligibility and will participate in either a maximal effort 2-day testing protocol or a submaximal effort protocol. A submaximal effort protocol has been included as an option as community feedback indicated unwillingness from some people due to the potential symptom flare-up predicted with the maximal protocol.

Effort testing on a bicycle ergometer is required to accurately determine the HR and RPEs at ventilatory threshold (VT) or 10% below the VT for each participant. This 10% below VT has been posited as a preferred limit to stay below to avoid symptom flare-up [23] and will be employed in this study for all 3 arms.

The 2-day maximal effort testing protocol is a smaller study running concurrently at the University of South Australia looking to confirm the differing physiological responses demonstrated by people with ME/CFS due to postexertional malaise during repeat exercise testing when compared to healthy controls. The 2-day maximal effort testing protocol will involve attending the university on 2 consecutive days and participating in the same testing protocol on a cycle ergometer wearing a gas analysis mask. A subjective questionnaire regarding fatigue status will be completed, followed by a 10-minute period of rest while wearing an HR monitor. The incremental exercise test on the bike will progress from very light to maximal exercise over a period of 8 to 12 minutes. The test starts with a 5-minute, 40 watt warm-up, during which rate of HR increase is assessed. Following this 5 minutes, the test increases using a ramp protocol, with increases of 5 watts every 20 seconds (so 15 watts per minute on average). The test finishes when the participant reaches volitional exhaustion. Both testing sessions will be conducted at the same time of day.

The submaximal effort testing alternative will involve attending the university on 1 occasion. Participants will complete 1 test on a cycle ergometer while wearing a gas analysis mask. Participants will exercise on a cycle ergometer commencing at a work rate of 25 watts and increasing by 15 watts each minute until a HR of 85% of age predicted maximum is achieved (208–[0.7×age]) [24]. The test will be terminated prior to this point if participant reaches voluntary exhaustion or reports an RPE of 19 or higher, respiratory exchange ratio of >1.15, or a plateau in HR or VO_2_ with increasing work rate. Recommended exercise intensity will be limited to an HR corresponding to 10% below measured VT or in the case that VT is not adequately detected in the exercise test then below 70% of age-predicted HR max.

### Health-Related Outcomes

#### Overview

There will be at least 4 weeks between effort testing and commencement of the intervention with 2 weeks being stable at a pretest pattern of symptoms/activity. Participants may require longer recovery before commencing the intervention.

All health-related outcomes will be measured at 3 time points: baseline, 6 months after baseline at the end of the intervention, and at 6-month follow-up after the intervention has been completed. In addition, accelerometer, inclinometer, and continuous captured HR will be measured during week 6, week 12, and week 18 of the intervention. The Karnofsky Scale [25] will be collected more frequently, with the research assistant recording the Karnofsky Scale value each time telephone contact is made with a participant.

Health-related outcomes have been chosen after extensive consultation with the stakeholder advisory group which included people living with ME/CFS. Outcomes have been selected to first provide data on the primary health-related outcomes (ie, physical activity and allostatic load:11 variables). Outcomes have also been selected to cover client-centered outcomes such as use of time, quality of life, and functioning. These outcomes are of utmost importance to the people with ME/CFS and capture the broader International Classification of Functioning, Disability, and Health framework concepts of activity limitations and participation restriction [26]. Finally, outcomes have been selected to capture the vast range of symptoms reported by people with ME/CFS to ensure the levels are monitored alongside the physical activity changes to ensure safety and investigate net benefit to the participants.

#### Adherence Diary

All 3 groups will be provided with a pen and paper adherence diary to complete daily for the 6-month duration of the intervention. The pacing only group will document adherence to pacing protocol and HR achieved (yes/no) and RPE limit achieved (yes/no). The conventional physical activity and video games groups will document minutes of activity, specific day, HR achieved (yes/no), and RPE limit achieved (yes/no).

#### Sociodemographic Questionnaire

A sociodemographic questionnaire developed for the purpose of this study will capture data such as age, gender, education level, working/volunteer status, income, and duration of conditions.

#### Symptom History Questionnaire

A questionnaire developed for this study will capture all symptoms related to ME/CFS and also the relationship between symptoms and activity to assist in the pacing and activity protocol prescription for each individual.

#### Accelerometer

GENEActiv (Active Insights Ltd) accelerometers will be worn on the nondominant hand for 7 days during each testing period. Accelerometers will be worn for 24 hours per day but will be removed for bathing and swimming activities. Participants will complete a log to report periods of nonwear time and sleep/nap times.

#### Inclinometer

ActivPAL (PAL Technologies Ltd) inclinometers are matchbox-sized devices that measure position relative to gravity. They are worn on the front of the thigh attached with hypoallergenic adhesive tape for a period of 7 days and report the minutes spent sitting, standing, and walking. Inclinometers are the current gold standard measurement of sitting/reclining time, a measure of sedentary time.

#### Multimedia Activity Recall for Children and Adults

The Multimedia Activity Recall for Children and Adults [27] is a computerized self-report 24-hour recall tool that allows detailed recording of time use behaviors that has been tested on various populations including adults and those with ME/CFS in a recent study conducted by several of the authors. Some participants with ME/CFS report some cognitive strain and burden completing more than 1 24-hour recall intervention over the telephone at once, so for this study, 4 interviews will be conducted each on separate days (during the week that the accelerometer is being worn) capturing at least 1 weekend and 1 weekday.

#### Height, Weight, and Waist Girth

Weight will be measured using the TANITA BC-418 bioelectrical impedance analysis scale. Participant weight will be taken with participant barefoot and clothed in lightweight clothing only. Height will be measured using the ECOMED seca 284 stadiometer. Two measurements will be taken for both weight and height. If the second weight measurement differs by >1%, a third measurement will be required. Similarly, if the second height measurement is >5 mm different then a third measurement is required. An average of the measurements will be obtained (2 measures) or a median (3 measures). Body mass index (BMI) will be calculated. Waist girth will be measured using an anthropometric tape measure and following standardized protocol by an accredited exercise physiologist.

#### Blood Pressure

Blood pressure will be taken using standard procedures and a sphygmomanometer while participant is sitting or reclining (same position each time). Systolic and diastolic blood pressure will be recorded.

#### Heart Rate Variability

RR intervals will be recorded during 5 minutes of supine rest prior to the exercise test using a personal HR monitor (RS800CX, Polar Electro Oy). RR interval data will be downloaded to Polar Protrainer 5 software (Polar Electro Oy) where artifacts or ectopic heart beats are removed through Polar’s automatic filtering. Data will then be exported to heart rate variability (HRV) analysis software (Kubios HRV Analysis version 2.0 beta 1, The Biomedical Signals Analysis Group) where any remaining artifacts or ectopic heart beats can be removed and the final 2 minutes of recording analyzed for the HRV time domain variable of interest (root mean square of successive differences).

#### Allostatic Load

Blood will be taken after fasting to collect data on insulin and glucose, total cholesterol and high-density lipoprotein (HDL) (so we can calculate low-density lipoprotein [LDL]), C-reactive protein (CRP), and adrenaline and noradrenaline. These measures cover both the primary mediators and secondary outcomes of the AL. Other measures used to calculate AL will include HR variability, BMI, waist girth, and blood pressure.

The AL index will be calculated by determining whether, for each participant, their variables are in the healthy zone (coded 0) or the unhealthy zone (coded 1) and then summed to provide a composite AL index. Some variables will be coded based on clinical cut points; the remaining variables that do not have accepted clinical cut points will be analyzed by calculating the upper 25th percentile (code 1), except HRV where the lowest quartile will indicate risk (code 1). The following criteria will be used to determine the risk zones based on previous studies or published guidelines:

- BMI >30 kg/m^2^ = 1
- Waist circumference >94 cm (men) and >80 cm (women) = 1
- Fasting lipid profile:
  - Total cholesterol ≥5.2 mmol/L=1
  - HDL <1.00 mmol/L=1
  - LDL ≥3.4 mmol/L=1
- Fasting glucose >5.5 mmol/L=1
- Systolic blood pressure ≥140 mm Hg or diastolic blood pressure ≥90 mm Hg = 1
- CRP >3.00 mg/L (cardiovascular risk cut point) = 1
- Adrenaline (highest quartile) = 1
- Noradrenaline (highest quartile) = 1
- HRV (lowest quartile) = 1

Prescribed medication will be taken into account so that an individual is given the value 1 (indicating health risk) for diastolic and systolic blood pressure if they used blood pressure lowering medication and for HDL cholesterol if they used lipid lowering medication. The maximum value AL=12, minimum=0.

#### Diet Diary

A pen-and-paper 3-day food diary will be completed capturing all food and drink consumed during the dates including sweets, snacks, nibbles, sauces and dressings, water and supplements (vitamins). The diet data will be analyzed using the FoodWorks software (Xyris).

#### Body Temperature

Body temperature will be measured using an electric thermometer. This will be measured to assess if there is an acute inflammatory response (fever). In addition, participant will also be asked to self-report presence of acute infection during testing periods.

#### Karnofsky Scale

The Karnofsky Scale [25] is used to assess the current abilities of the participant based on a numerical scale from 0 to 100. These scales allow a quick assessment of the participant’s function.

#### Chalder Fatigue Scale

The Chalder Fatigue Scale [28] is one of the most widely used measures for assessing physical and mental symptomatic fatigue experienced by ME/CFS patients. Four response options are available, ranging from “less than usual” to “much more than usual.” The Likert system for scoring was used (0, 1, 2, 3), with a total possible score ranging from 0 to 33. A higher score indicates more fatigue.

#### Cognitive Failure Questionnaire

The Cognitive Failure Questionnaire [29] assesses self-reported deficits in attention, perception, memory, and motor functioning. The questionnaire measures the frequency of everyday cognitive failures or lapses by asking participants to rate how often they make mistakes on a 5-point Likert scale, from 0 (never) to 4 (very often). The instrument produces a global “cognitive complaints” score (ranging from 0-100), with higher scores indicating more cognitive failures.

#### Pittsburgh Sleep Quality Index

The Pittsburgh Sleep Quality Index [30] is commonly used to collect data regarding subjective sleep quality, sleep latency, sleep duration, habitual sleep efficiency, sleep disturbances, use of sleep medication, and daytime dysfunction. The seven component scores are rated from 0 to 3, with 0 being no difficulty and 3 indicating severe difficulty. The composite score of the 7 subscale scores provides a global score ranging from 0 to 21, where higher numbers indicated poorer sleep quality.

#### Short Form 36 Questionnaire

The Short Form Health Survey [31] is a 36-item patient-reported survey of patient health commonly used as a measure of quality of life. The questionnaire was developed at RAND Health as part of the medical Outcomes Study.

#### Cambridge Neuropsychological Test Automated Battery

Cognitive performance will be assessed at each visit using 8 well-validated tests assessing reaction time, attention, memory, and executive function from the Cambridge Cognition, suitable for people living with ME/CFS. Tests include motor training, simple and 5-choice reaction time, spatial working memory, pattern recognition memory immediate and delayed, one touch stockings of Cambridge, attention switching task, and rapid visual processing. The order that tests are delivered has been determined with key experts in cognitive testing at Cambridge Cognition so longer tests are separated with shorter tests. This will ensure adequate breaks between testing occur. All testing is automated on a tablet to reduce administration error between participants. Furthermore, testing will take place in a small quiet clinic room with a bed available if participant needs to lie down and rest for administration or in between tests. Snacks and water will be available to participants throughout the cognitive testing.

### Adverse Events

A comprehensive adverse events reporting protocol has been developed for this study based on the stakeholder group feedback and previous literature. An adverse event (AE) will be defined as “any clinical change, disease, or disorder experienced by the participant during their participation in the trial, whether or not considered related to the intervention studied in the trial.”

AE data will collected (1) during every preplanned phone contact between the participant and the research assistant, (2) if the participant contacts the research assistant to report an AE, and (3) during the 3 testing sessions (baseline, 6 months, and 12 months). Questions will be asked about the following 3 categories to encourage the participant to report AEs: any new comorbid medical conditions reported if not previously reported at baseline, any events for which the participant consulted their general practitioner or other medical advisor or took medication, any other events that might have affected the health status of the participant (eg, increased work stress).

The research assistant will have knowledge of participant allocation (ie, control or active intervention group) and as such will be able to use any AE data reported to provide activity counseling and advice regarding the intervention. If an AE is reported, participants will be asked whether they consider the event to be directly caused by the intervention.

A serious adverse event (SAE) will be defined as one of the following outcomes:

- Death
- Threat to life (ie, an immediate, not hypothetical, risk of death at the time of the event)
- Required hospitalization except for elective treatment of a preexisting condition
- Increased severity and persistent disability, defined as (1) severe (ie, significant) deterioration in the participant’s ability to carry out their important activities of daily living (eg, employed person no longer able to work, caregiver no longer able to give care, ambulant participant becoming bed bound) and (2) symptom and disability persistent (ie, of at least 4 weeks continuous duration)
- Any other important medical condition which, though not included in the above, might require medical or surgical intervention to prevent one of the outcomes listed

For any AE established as serious, a medical specialist member of the stakeholder advisory group will be asked to establish whether or not the event was a serious adverse reaction based on treatment allocation information. A serious adverse reaction will be considered to be a reaction to the trial. Both medical specialist and participant attribution will be reported.

A nonserious adverse event (NSAE) will be any health event that was not categorized as an serious AE or serious adverse reaction. NSAEs will be rated as mild, moderate, or severe by both the participant (at the time of reporting) and a medical specialist. The duration of any increases in severity or disability from these events will also be recorded.

Each NSAE will be ascribed a body system (gastroenterological, neurological, etc) by a health practitioner team member. NSAEs attributed to ME/CFS (ie, considered to be a symptom of ME/CFS) will be put into a separate category. To monitor disability, each time the research assistant calls a participant they will be requested to self-report their disability on the Karnofsky disability scale.

### Testing Considerations

All equipment and testing facilities will be perfume-free (or as low as possible) as many people with ME/CFS also report multiple chemical sensitivities. Testing facilities will provide footstools and beds to rest. Rest periods will be provided at the request of the participant during the testing sessions to reduce fatigue. Paper questionnaires can be sent home with participants to be completed at their own time and mailed back to spread the effort. Cambridge Neuropsychological Test Automated Battery testing can be paused at one point to allow a rest period. Breakfast/food will be provided after the fasting bloods have been taken.

### Analysis Plan

Exit questionnaires will be audiorecorded, transcribed verbatim, and sent back to participants for member checking. Themes will be extracted and summarized from interview transcripts.

Analysis of continuous data to assess preliminary evidence of benefit will include examination of mean differences and confidence intervals ranging from 75% to 95%. Regression analysis will be used to explore preliminary evidence of a relationship between allostatic load and physical activity. The alpha level will be set at 0.2, an appropriate level for a pilot studies [32]. Categorical questionnaire data will be analyzed using chi-square analysis. As this study is a pilot study, any inferential statistics will be purely exploratory (ie, not powered to detect statistical differences).

## Results

A 6-month extensive consultative protocol development phase has been completed; 3 comprehensive rounds of protocol topics, comments, and plans for amendment occurred.

The first round focused on the following general topics: inclusion criteria, time frame between effort testing and intervention commencement, time and frequency of assessment, and the inclusion of continuous HR data collection. The Karnofsky Scale and alternative disability scales were discussed. The second round included discussion on the following broad topics: design modification to 3-arm trial, symptom history data collection, and the pacing and physical activity protocol. The third round involved discussion on the following topics: AEs protocol, adherence diary, and clarification of maximal and submaximal effort test procedures.

Virtual meetings allowed for interactive group discussion, and consensus was reached on all aspects of the protocol. All members of the stakeholder advisory group have endorsed the current protocol. Enrollment began in January 2017; as of publication, 12 participants were enrolled. Baseline testing is scheduled to commence in mid-2017.

## Discussion

### Summary

The proposed research will fill gaps in the existing literature by (1) providing only the second known study of symptom-contingent pacing as a strategy to increase physical activity in adults with ME/CFS, (2) providing the first data on feasibility and acceptability of using active video games as a strategy to increase physical activity in people with ME/CFS, and (3) providing longitudinal data regarding the relationship between allostatic load and physical activity in people living with ME/CFS.

Adults living with ME/CFS often suffer orthostatic intolerance, and as such, a physical activity modality that can be achieved in a sitting or reclining position is advantageous. In addition, many people living with ME/CFS report short bouts of activity to be easier to manage, hence an activity modality that allows short bouts of several minutes to then allows adequate rest addresses the consumer requirements. Active video games such as Nintendo WiiFit and Xbox Kinect have been demonstrated to demand low to moderate levels of physical activity in the elderly, even when sitting [18]. So playing these games while sitting/reclining/lying can still be useful. Active video gaming has been shown to be feasible and acceptable to other chronic health populations such as those with cerebral palsy and stroke and is commonly played sitting in these populations [17].

Evidence suggests active video gaming can improve the health and well-being of people with a variety of chronic and debilitating health conditions [17]. Despite the evidence to suggest active video gaming as effective and relevant, no studies have investigated the effect, feasibility, or acceptability of active video gaming in adults with ME/CFS.

### Conclusion

The results of this trial will provide the feasibility and acceptability data to suggest whether active video gaming can be pursued as a strategy to help people living with ME/CFS overcome physical inactivity. The pilot study will provide important data to guide future effectiveness trials and is intended to deliver safe, effective, evidence-based, consumer-driven physical activity interventions to the ME/CFS community.

## Abbreviations

AE: adverse event
AL: allostatic load
BMI: body mass index
CRP: C-reactive protein
GET: Graded Exercise Therapy
HDL: high-density lipoprotein
HR: heart rate
HRV: heart rate variability
LDL: low-density lipoprotein
ME/CFS: myalgic encephalomyelitis/chronic fatigue syndrome
NSAE: nonserious adverse event
RPE: ratings of perceived exertion
VT: ventilatory threshold
